# Gateways, Funnels, and Stackers: How People Hide Property Ownership Through Offshore Structures

**DOI:** 10.1111/1468-4446.70075

**Published:** 2026-01-09

**Authors:** Kristin Surak, Johnathan Inkley

**Affiliations:** ^1^ Department of Sociology London School of Economics London UK; ^2^ Department of Sociology Cambridge University Cambridge UK

**Keywords:** elites, inequality, offshore, real estate, tax havens, wealth

## Abstract

How do wealthy individuals use offshore financial structures like shell companies to protect personal assets? And how is such offshore wealth structuring itself variably organized? Moving beyond conceptualizations of offshore as concerning only individual tax havens, this article investigates offshore wealth structuring as a fundamentally relational practice to supply the first systematic image of the patterns between two key layers of offshore structures within a specific asset class. We analyze the overseas entities that hold expensive residential properties in the UK to make three contributions to debates around offshore. First, we identify a specific regional offshore circuit in its flows and magnitude by isolating two key layers, namely the *entry layer*, which is used to connect into the UK property market, and the *action layer*, which is used for the actual or projected appearance of managing the offshore structure. We next examine the interstices between these layers to reveal three patterns of offshore formations. These we term *global funnel*, *selective gateway*, and *self‐stacker*, and we discuss their implications. Finally, we offer indirect evidence of which jurisdictions people are more likely to choose for “brass plate” incorporation and which they employ for more complicated structuring, either in actuality or in appearance, which has implications for policymaking. By identifying significant variation in the interstitial patterns between jurisdictions, we not only pinpoint which jurisdictions are used in relation to others and to what extent, but also provide indirect evidence of how they are used differently and discuss why. Our findings supply a pioneering analysis of the scope, scale, and interstitial formations of the offshore structures that wealthy individuals use to hold personal property.

## 1 Introduction

In the everyday imaginary, terms like shell companies, tax havens, offshore finance, and offshore wealth structuring evoke pictures of peripheral places that attract shady characters, facilitate illicit dealings, and enable wealth to hide. Academic work, however, has revealed that offshore does far more. Research on offshore financial flows estimates that as much as 10% of global GDP and 8% of global household wealth moves through offshore formations and makes use of the legal and financial advantages that they afford (Zucman 2015). In fact, it would be unusual for a sizable multinational corporation *not* to employ the regulatory arbitrage that offshore structuring facilitates for the protection of assets (Palan and Phillips 2021). Offshore structuring is not simply tangential to global capitalism, but constitutive of it (Palan 2003). As Maurer (2008: 160) describes, “Far from a marginal or exit backwater of the global economy, offshore in many ways is the global economy.”

Yet we know relatively little about how such offshore structuring works in practice. Academic debates continue about what counts as offshore, where is offshore, and its actual scale and patterns in situ. Qualitative research has made the important strides, providing productive insights into descriptive questions of how and why. However, quantitative research has remained some lengths behind it, traditionally stymied by the paucity of data. As a set of leading economists in the field recently lamented, we still have a poor understanding of even basic questions about offshore financial centers and global wealth (Alstadsaeter et al. 2017). For example, recent work has investigated the first layer of offshore structures used to hold UK property (McKenzie and Atkinson 2020; McKenzie et al. 2024; Bourne et al. 2023; Collin et al. 2022; Bomare and Herry 2022), but data limitations have obstructed the investigation of deeper offshore structuring patterns and we still have much to learn. How do wealthy individuals leverage multiple offshore layers to hide their ownership of real estate assets? What differences are there in how the jurisdictions connect to each other and how they operate?

In this article, we draw on newly available data to investigate how wealthy individuals use offshore structures to hold residential real estate. In doing so, we go beyond conceptualizations of offshore as about particular places—namely individual tax havens in isolation—to investigate offshore as fundamentally about the interstices between legal matrices. As such, analyzing offshore requires dissecting the relationships *between* jurisdictions. To do this, we systematically capture two key layers of a specific offshore circuit in both its flows and magnitude. These are the *entry layer*, which is used to enter into the jurisdiction and directly hold the property, and the *action layer*, which is used for the actual or affected management of the structure. We unpack the connections between these layers to supply an interstitial analysis of a regional offshore circuit in practice. We identify the jurisdictions chosen, patterns in how they link to each other, and possible differences in what they do and why. The analysis reveals three distinct patterns of offshore formations: some jurisdictions are used by entities around the world, others are used only by entities within the same country, and some combine the two. We term these *global funnel*, *self‐stacker,* and *selective gateway*, and we discuss the implications of each. Finally, we supply indirect evidence of which jurisdictions people are more likely to choose as places for “brass plate” incorporation services and those they are more likely to select for more complicated structuring or for affecting the appearance of greater action and effective “substance.” These differences in the relationships *between* offshore layers and how people use them have implications for policymaking. Our findings supply a pioneering analysis of the scope, scale, and key formations of how wealthy individuals structure particular personal assets by identifying the distinctive patterns between two key layers of the offshore structures that they use to hold expensive residential property in the UK.

### 1.1 Offshore Structuring

If offshore structuring appears murky to the general public, it can as well to academics. Indeed, there is still no consensus on just *what* constitutes offshore. Casting the widest net are scholars, often economists, who define offshore as all wealth or assets held by individuals outside their country of residence (De Simone et al. 2018; Alstadsaeter et al. 2017; Johannesen and Zucman 2014). This, for example, is the definition employed by the EU Tax Observatory in much of its work, which designates *all* foreign transactions—or transactions involving foreigners—as offshore (Alstadsaeter et al. 2017; Alstadsaeter et al. 2022, 2024; see also the definitions employed in the Atlas of the Offshore World^1^). This approach can also be found in studies of Eurodollars and the Foreign Account Tax Compliance Act (FATCA) that treat all transactions in US dollars made outside the United States as offshore by default (Binder 2013; De Simone et al. 2018). In these cases, offshore equals simply anything that is outside the country at hand.

Others, often employing political economy or sociological approaches, define offshore in terms of what it does. At heart, offshore supplies a “zone of freedom from regulation and accountability” (Harrington 2016, 130). The financial activity that happens in such places is “fictional”—that is, the activity or asset is treated “as if” it was there (Harrington 2016; see also Palan 2002). Enabling these plays is a strategic legal bifurcation that splits a state's jurisdiction into heavily regulated and lightly regulated parts, with the latter servicing the offshore sector. Common traits of such places include English or US common law, which afford useful forms of legal flexibility, and regulatory authorities that are willing to allow professionals to lay out acceptable business practices. In many cases, they aim to create “calculated ambiguity,” which enables actors “to give diametrically opposed but legally valid answers when responding to the same question from different audiences” (Sharman 2010, 2). As Haberly and Wojcik (2014: 5) describe, “What defines offshore finance…is less the jurisdiction within which transactions are booked or conducted, than their conduct in a networked transnational legal space produced by the lack of a clear legal basis for multinational activity.” The upshot facilitates arbitraging multiple legal systems or playing in the interstices between jurisdictions (see Palan and Phillips 2021; Hoang 2022). With legal bifurcation and arbitrage as its fundament, offshore is better seen not a specific geographical location that money goes supposedly *into*, but as a matrix of juridical areas in which standard regulations are withdrawn or rewritten to enhance the legal and financial benefits, which emerge at the interstices between offshore layers, for its end users (Palan 2003). Notably, these benefits are made available to foreign entities, rather than local ones. That is, the legal carveouts and financial advantages are targeted at those accessing the system from the outside (Harrington 2025). Onshore, by contrast, operates based on the coincidence of territorial and juridical presence (Haberly and Wojcik 2014). As such, offshore cannot be offshore to itself; the result would be onshore.

To investigate the relations and interstices that define offshore requires analysis into the different layers of offshore structures. Offshore ownership, for example, is often nested, with one entity owning another—or even bundles of entities—resulting in structures with multiple layers. However, these deeper layers are extraordinarily difficult to probe, which is one of the functions of offshore in the first place. To date, the best work in this area has been carried out by network scholars. One set has explored how elite individuals structure their wealth by mining data leaks, such as the Panama Papers, Paradise Papers, and Pandora Papers, to identify differences in transnational patterns of offshore usage. Analyzing the ICIJ Offshore Leaks Database as a snapshot of multiple layers in time, Chang et al. (2021) show that the offshore financial system functions as a “scale‐free network” in which connectivity can be concentrated in a small number of nodes that are also chokepoints for the system. Others have focused on how multinational firms use offshore structures by analyzing the ORBIS dataset, which contains information on more than 600 million companies. Research on the global ownership chains underlying multinational corporations has revealed that some jurisdictions serve as “sinks” that store capital while others operate as “conduits” that facilitate the movement between sinks and other countries (Garcia‐Bernardo et al. 2017). Others have distinguished between “standalone” jurisdictions that appear at the end of chains and “in‐betweeners” that offer flexibility for rerouting profits (Phillips et al. 2020; Palan and Phillips 2021). At the same time, work with these depth‐rich datasets confronts important limitations. The ICIJ Leaks are an abundant sample, but a biased one. The Panama Papers and Paradise Papers contain information from single firms, for example, and it remains impossible to assess how representative they are of the global field. The ORBIS dataset, too, includes sampling biases that are significant but difficult to control: it usually has better quality information for high‐income countries than low‐income ones and excludes many key offshore locales including small jurisdictions and subnational jurisdictions that are popular for incorporating shell companies. Investigations of depth often face a tradeoff in generalizability.

In an attempt to square the circle, our analysis combines the insights of the strands above to investigate the connections between two key layers of offshore structures for a specific asset class, namely UK residential property. We take as our departure point the economistic approaches that define offshore as concerning entities established outside the country at hand. People operating in this space are driven by the legal and financial advantages gained by going to the extra effort of establishing foreign entities to enhance their business interests or protect their personal wealth. As such, we identify specific offshore locations based on the structuring choices that people make in practice rather than categorizing them based on politicized offshore lists. We then incorporate the insights of political economists and sociologists who stress that the advantages that these structures offer inhere in the interstices between different legal regimes (Sharman 2010; Harrington 2016; Palan 2003). To investigate this, we take an interstitial approach that identifies basic patterns in the jurisdictions that people choose to illuminate the common configurations between two key layers of the structures.^2^

Studying offshore in this way means that we do not treat offshore as self‐same with tax havens. Though the two are often conflated, the difference is important. The term “tax haven,” along with its correlate “offshore financial center,” is typically used as a label that is applied to places offering preferential tax regimes and hosting financial services aimed at benefitting foreign entities. If a distinction between them is made, it is usually that the tax haven label emphasizes the former and offshore financial center emphasizes the latter (for a discussion, see Palan et al. 2010). Academic research commonly identifies such places by combining various tax haven and offshore financial center lists to develop compilations or “consensus lists” of jurisdiction that simply *are* offshore. As others have pointed out, these exercises can end up reproducing the vested interests and power politics involved in determining what jurisdictions get counted in the first place (Jansky et al. 2022), and the most powerful players like the US, UK, and Switzerland often remain excluded from them (Sharman 2010; Haberly and Wojcik 2014). More importantly for our present analysis, labels like “tax haven” and “offshore financial center” misconstrue the fundamentals of offshore and how it operates. As described above, offshore is not about a singular essence that a jurisdiction possesses. Rather, it is fundamentally relational: the *where* of offshore is always a function of standpoint. The UK, for example, may be cast as an offshore financial center that offers services privileging foreign entities, but it cannot be directly offshore to itself. Therefore, we employ the term “offshore” as a general descriptor to capture relational plays rather than as a label applied to single, context‐independent places.

We know from prior research that offshore—or the variety of matrices sometimes referred to as “the offshore world”—is not homogeneous. To a notable extent, it operates as an ecology of options that are useful for doing different things. Jurisdictions may try to carve out niches by specializing in or developing their reputations around particular services (Palan et al. 2010). The Cayman Islands are a top choice for foreign investment funds while Bermuda has transformed itself into a popular option for global insurance and reinsurance industries. Although many places offer fast and easy company incorporation, the British Virgin Islands (BVI) has successfully cultivated a reputation as the go‐to location globally for shell companies (Sharman 2012). In addition, how multinational corporations—often concerned with issues of investment, trade, and corporate taxes—employ offshore structuring is substantially different to the personal wealth structuring of individuals, who are more likely to be concerned with inheritance, divorce, and various forms of personal taxes.

A further point of variation is the difference between services that are simply “brass plate” and those that supply more “substantive” activity (Sharman 2009; see also Sharman 2011). Brass plate offerings enable actors located anywhere to do things such as set up legal entities like shell companies swiftly and cheaply. These services require only a light physical presence on the ground and operate largely as a tool in the toolkit of professional service providers located elsewhere (Morriss and Ku 2024; see also Palan et al. 2010, Sharman 2009). In the British Virgin Islands (BVI), for example, it is possible establish a corporation in less than 48 h for under $1000 from anywhere in the world. Substantive activity, by contrast, goes beyond simply legal entity formation and requires financial and legal professionals to carry out activities on site. This professional class will have the training and licensing to serve on boards of directors, offer complex legal advice, and provide other facilities involved in financial structuring (Morriss and Ku 2024; see also Sharman 2009). Unlike jurisdictions that ride on brass plate offerings, those that develop reputations for offering substance are more likely to attract a sizable base of not only private clients but also business or institutional clients (Sharman 2009).

This is important because even if an offshore entity's asset isn't in the jurisdiction where the entity is located, securing certain legal advantages can depend on showing that substance or management is—or is in effect—occurring there. One indica of this is the entity's correspondence address. This address tells authorities how they can reach the individuals behind the entity, which may be the actual owners but are more frequently the service providers representing them and who created the structure. Ostensibly, the correspondence address will match the location of the individuals managing the structure, but it need not do so. Whether in fact or in appearance, however, it can be read by authorities as an indication of “substance,” or the place where management activity occurs.

If we know that the functional ecology of offshore is varied, research identifying such patterned differences has faced challenges. Qualitative work can isolate key types and locations but confronts limits when establishing their scope, scale, and patterns of operation. Some quantitative studies have offered basic descriptions of which countries and territories are chosen for establishing first‐layer shell companies (McKenzie and Atkinson 2020; Bomare and Herry 2022; Collin et al. 2022; Johannesen and Zucman 2014), but they are limited to constructing popularity ranks and measuring changes in selections. Without thicker data, they remain unable to identify differences in how the jurisdictions operate as a part of a multi‐level system or which jurisdictions people employ for brass‐plate work and which they select when seeking greater substance. Our analysis contributes to these efforts by analyzing the connections between two key layers of offshore structures used to hold residential property.

In doing so, we are not arguing that we identify a scaled‐down representation of a wider “offshore world.” The ecology of offshore is not a single landscape that exists independently of a particular standpoint but is fundamentally relational. Offshore operates based on the advantages generated at the interstices of legal systems meaning that the benefits gained will hinge on the web of juridical obligations that impact a person—whether a human being or a legal being—based on their locational and membership particulars. Whether and how Malta can serve as an offshore financial center for an individual, for example, will depend on whether the person is a Maltese citizen, a citizen of another EU country, or a third‐country national, as well as where they are primarily located or spend time, on top of the types of service at stake. Offshore is positional by definition.

As described above, offshore encompasses a range of services targeted at accomplishing different tasks for different clientele. To control for this substantial variation, we focus our analysis on a particular asset class—expensive residential real estate—and how it is “wrapped,” or held through a foreign entity, such as a holding company or a trust, rather than owned directly. We analyze the UK property market, which has been identified as one of the top locations for offshore property holdings, constituting upwards of 20% of all cross‐border real estate investments globally (Bomare and Herry 2022). Research to date has been able to identify only the first layer of offshore entities holding property in this market with systematicity. McKenzie and Atkinson (2020) combine two datasets to identify the primary first‐layer jurisdictions used to hold UK property between 1994 and 2014, revealing that BVI and Jersey predominate, followed by Guernsey. Bourne et al. (2023) focus on offshore‐held properties as one element of “unconventional residential property” in London to show that those owned via offshore entities are more likely to be located in the center of the city. Other analysts have combined UK data with international datasets to trace a selection of extended ownership chains. Johannesen et al. (2022), for example, identify a small set of second‐layer corporate owners by using ORBIS, but do so only for cases in which a first‐layer holding structure is in the UK rather than offshore to it. Bomare and Herry (2022) employ the ICIJ Leaks to peer more deeply into ownership chains, which enables them to identify beneficial owners in 2.8% of the cases in which the first‐layer entity is offshore. However, their focus is on the beneficial owners and they put aside questions about how the offshore structures themselves are layered. Also using ICIJ Leaks to trace a portion of ownership chains, Collin et al. (2022) examine the impact of the announcement of a beneficial ownership registry on the UK property market and find that new purchases wrapped by first‐layer companies in tax havens fell substantially. However, they too do not examine multi‐layered structuring patterns.^3^

This literature has furthered our understanding of offshore real estate holdings, yet important questions remain unanswered. Most work agglomerates all properties, which means that the structuring patterns of multinational corporations, for example, are mixed in with those of individuals holding personal assets. Furthermore, analysts capture either only the first offshore layer or only the second layer that is offshore to UK companies, or else they focus on identifying beneficial owners through data leaks. To date, none have examined the relationships between offshore layers—a promising area of analysis made more accessible through newly available data, as discussed below. If offshore operates at the interstices between legal jurisdictions, what sorts of relationships do we find between the layers?

By investigating this, we are able to systematically answer fundamental questions about the locations, relations, and scale of two key layers of offshore holdings for a given asset class, as well as identify three distinct formations of offshore usage that appear in practice. These we term *global funnel*, *selective gateway*, and *self‐stacker*. We also offer an indirect analysis of the scope and scale of brass plate versus substance usage within them. The upshot is the clearest image to date of an important regional circuit of offshore flows, namely the two key layers of the complex ecology of offshore structures employed in holding expensive residential properties in the UK.

## 2 Methods

Our analysis draws on a dataset of the beneficial owners of UK property that we created by combining three publicly available data sources. The first two are the *Free Company Data Product* and the *People with Significant Control* (PSC) register, which are maintained by the Companies House, the UK Government agency in charge of company registrations. We combined these with the *Overseas Companies that Own Property in England and Wales* dataset (known as OCOD, based on its former name) maintained by the UK government's HM Land Registry. The result is a dataset that mirrors the information that should be contained in the Register of Overseas Entities (ROE), a non‐public government register introduced after Russia's invasion of Ukraine to identify the owners of overseas entities holding UK property.

The *Free Company Data Product* contains a list of all entities currently active in Companies House, including information on their names, registration numbers, registered address, and country of registration. The PSC register contains a list of all entities reported to Companies House as having “significant control” over an entity. Of interest here are the entities registered as beneficial owners of overseas entities that hold UK properties. The Economic Crime (Transparency and Enforcement) Act of spring 2022 mandated all overseas entities to provide information on their beneficial owners. Full compliance was required by January 2023, and as of May 2024 the overseas corporate owners of 83% of such properties had complied. These beneficial owners can be an individual, a corporate entity (usually another company), or a legal person (usually government bodies, such as the tourist board of a foreign government). There are a small number of cases where a corporate beneficial owner is also listed as an overseas entity itself and individual beneficial owners of the top‐level entity can be identified recursively. Information on the land and property titles held by these overseas entities is contained in OCOD. The dataset includes basic information related to each property title. For the present analysis, we use the property's address and its listed “proprietors” to ascertain its location, as well as the name and location of its corporate owners. Notably, the register includes information only for England and Wales and not Scotland or Northern Ireland. For concision, we use “UK” throughout this article as a shorthand for the subset of England and Wales—which accounts for approximately 90% of all real estate transactions in the UK (Bomare and Herry 2022)—and is a convention used elsewhere by analysts employing these data (e.g., Bourne et al. 2023; Johannesen et al. 2022; Bomare and Herry 2022). In the case of residential properties, we have information on purchase price for 48% of properties.

We merged the three datasets above based on the name of the entities holding properties and the country of registration. Across the data sources, however, neither the name of the entities nor the country of registration was inputted in a standardized manner. Name standardization was carried out by removing common company suffixes. Country standardization required mapping country names to the aliases for them that appear in the data. For example, if the jurisdiction of an entity is given as “Delaware,” it was mapped to “U.S.A.” With this approach, we matched 83% of all titles listed in OCOD to a corresponding entity (or set of entities in the case of multiple proprietors) in the *Free Company Data Product* and the PSC register. The cases where titles could not be matched result from non‐compliance, poor quality data, or a small number of cases where registration is not necessary (see The Law Society 2022). More complex country standardization was employed to determine both the nationality of individual beneficial owners and the country of registration of corporate beneficial owners. Lack of consistency in how the information was inputted presented further challenges and required us to use fuzzy matching. For example, we found 95 different ways that the United States could be identified for this variable, including “U.S.A.,” “New York,” or even “The Delaware General Corporation Law (Title 8, Chapter 1 Of The Delaware Code).” For Hong Kong, 22 values were identified, including “Hkicpa.” Where fuzzy matching could not be employed, we used manual matching. From matching these three datasets, we produced a network of land titles, entities, and individuals that are connected by forms of ownership. We completed our data matching for all entities through July 2024, and the combined datasets enabled us to identify 31,000 overseas entities that own properties corresponding to a total of 77,000 Land Registry ownership titles in the UK.

Our wider dataset of all wrapped properties encompasses a range of property types—including rental units, business properties, parking lots, and corporate headquarters, among others—and the differences between them can impact ownership and wrapping styles. For example, an investment fund holding rental real estate for investment purposes is likely to adopt a different wrapping style to an individual wrapping a personal residence, who is likely to be concerned about separate array of tax and legal issues. To keep from muddying the waters by agglomerating offshore structuring patterns employed by different types of actors for different purposes, we focus on expensive residential property as this is likely to be associated with an important modality of offshore structuring, namely that of individuals structuring their personal wealth (see Fernandez et al. 2016).

We identified residential properties with an algorithm developed by Bourne et al. (2023). To pinpoint those meeting our price threshold, we took properties with an identifiable transaction price and date and modified the price to account for house price inflation. For this we used the UK Land Registry's granular House Price Index, allowing us to accurately account for house price trends at the local level.^4^ In the cases where the property classification algorithm suggested the property title contained several properties, we used a price threshold scaled by the number of properties in the title. This gave us a total of 6120 property titles for analysis, or approximately 8% of all the matched titles.

We analyze the patterns in the relationships between two key layers in our dataset by constructing three metrics. The two key layers are the *entry layer*, which is the first or lowest level of wrapping and used to enter into the UK property market, and the *action layer*, which is where real or projected action of more complex structuring takes place. We discuss these layers further in the Analysis section. Our first metric, *homeness*, attempts to capture the degree to which economic activity is genuinely occurring in a given jurisdiction. To do this, we measure the extent that the corporate beneficial owner and the entry‐layer entity are both registered in the same jurisdiction—that is, whether or not ownership chains are “stacked” in the same place. For example, if all entities registered in Country A have their corporate beneficial owner also registered in Country A, the homeness score is 100%. Conversely, if all entities registered in Country A report corporate beneficial owners registered elsewhere, the homeness score is 0%.

Our second metric, *dispersion*, assesses patterns in the overall usage of jurisdictions. Some jurisdictions have a history of providing financial services to individuals from a particular nation or set of nations. For example, Cyprus has long served as a financial center for Russian nationals (see Surak 2023). However, other jurisdictions attempt to capture business from across the globe. Our dispersion metric measures the degree to which a jurisdiction is used by “anyone and everyone” or by actors in specific places. A low dispersion score suggests that it provides offshore functions for actors from a small set of jurisdictions. A high dispersion score suggests that actors from a wide range of places use a given jurisdiction.

Our formula for dispersion employs what is known as entropy in the Information Theory literature (see Garcia‐Bernardo et al. 2017 for a similar application of entropy to unpack patterns of offshore structuring in the case of multinational corporations). Concretely, we define dispersion for a jurisdiction i as:

$$D_{i}=-\sum_{j}P_{ij}\text{ln}\left(P_{ij}\right)$$

Where j ranges over all jurisdictions, and $P_{ij}$ is defined as the percentage of entry‐layer entities established in jurisdiction i with an action layer owning an entity established in jurisdiction j.

Our third metric, *address alignment*, assesses whether there is a difference in the location of an entity's operations and the location of its official registration. For each action‐layer entity, we compare the jurisdiction where it is located with the jurisdiction given for its correspondence address. The *address alignment* score for each jurisdiction is the percentage of action‐layer entities registered in a jurisdiction that also have listed their contact address in that jurisdiction. A score approaching 100% shows that all align, which can be taken as a minimum condition of substance. A score approaching 0% would suggest instead that the jurisdiction is being used as a brass plate for registering an entity whose substantive functioning occurs elsewhere.

### 2.1 Analysis

By combining the datasets as described above, we are able see two key layers of overseas entities used to hold property in the UK. The technique of using an overseas entity to own or hold an asset, such as a property or another entity, is known as “wrapping.” In most cases within our data, the type of entity used for wrapping appears to be a holding company—effectively, a shell company—but in 15% of cases, we are able to identify trusts due to the presence of “trust” or “nominee” in the entity's name. Wrapping occurs from the top layer down. In a three‐tiered system, an actor will set up an entity at the third layer, which will then wrap an entity in a second layer, which will wrap an entity in the first layer, which finally wraps the property at the bottom. Our data, however, allows us to see only two key layers of wrapping, which are reported to the UK government. These we term the *entry layer*—or the layer where we find the offshore entity that is used for entering into the UK property market and wrapping the property itself—and the *action layer*, which is the layer of interest for enforcement purposes. At this layer is the beneficial owner reported to the UK government. According to UK law and reporting requirements, this is the layer where the action of a structure takes place.^5^ If, for example, ownership is divided in order to avoid reporting the ultimate beneficial owners to the UK government, UK law considers it to be happening at this layer.

**FIGURE 1:**
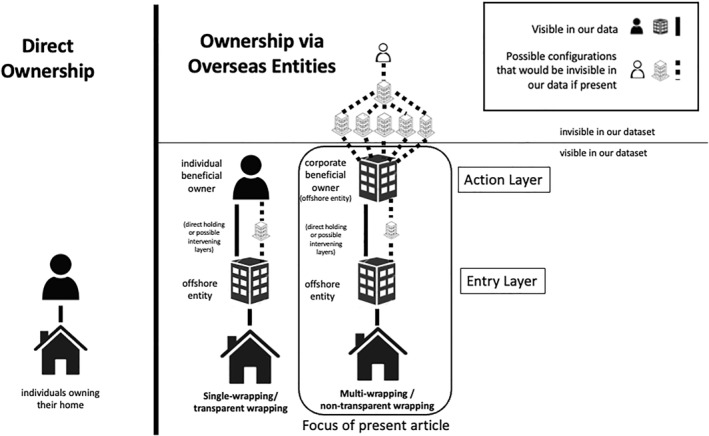
Direct and offshore ownership structures. It is possible that are there are intermediate structures between the entry layer and the action layer, as well as—in the case of corporate beneficial ownership—above the action layer. We suggest what this might look like in the above figure, though other configurations are possible.

Seen from the bottom up, our data allow us to view two configurations: transparent and non‐transparent wrapping. In the case of transparent wrapping (effectively single wrapping), the property is wrapped by an entry‐layer entity which is owned by an identifiable human being, known as an “individual beneficial owner.” In non‐transparent wrapping (effectively multi‐wrapping)—the focus of this article—the property is wrapped by an entry‐layer entity, which is subsequently wrapped further by an action‐layer entity, or “corporate beneficial owner” (Figure 1). In these cases, we are able to identify the jurisdiction that an actor chooses for their action‐layer entity and the jurisdiction of the entry‐layer entity that holds the property, but we are unable to ascertain information about the ultimate owner themself.

**FIGURE 2:**
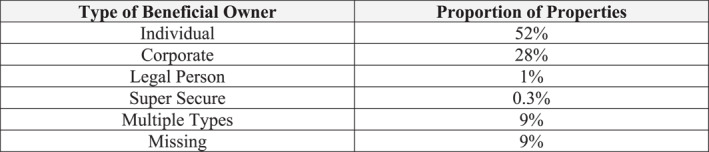
Type of beneficial owner reported for residential properties transacted for at least £1 million (2024). Authors' compilations.

In the bulk of the data—52% of cases—the wrapping has only a single layer of reporting significance. In such cases, the wrapping is effectively, transparent to the UK government and wider public and we are able to identify an individual beneficial owner (Figure 2), which we analyze elsewhere. In a further 19% of cases, the data are missing, multiple types of owners are given, the owner is categorized as a “legal person,” or the ownership is protected through a “super secure” option that reveals the identity of the owner to the British government but does not include them on the publicly available registry. In this article, we focus on the patterns of non‐transparent multi‐wrapping, that is, holding a property though at least two significant layers of entities which enables the ultimate beneficial owner or owners to remain hidden. We find this pattern in 28% of cases. The wrapping could go on for many layers, but we are only able to see the entry layer and action layer in our data, reported as the corporate beneficial owner to the UK government.^6^

We do not analyze why people choose to wrap, though several advantages can be gained. The two principal possibilities are privacy and tax benefits. Holding a property through multiple layers of entities increases the difficulty of establishing who is its ultimate beneficial owner (see Maurer 2013, 139). Individuals may wrap because they are apprehensive about governments, debt collectors, divorce lawyers, and others making claims on their assets and therefore want to shield their identity. Concerns about political stability and the rule of law in a person's home country or country of residence, too, can increase the desirability of hiding one's identity (Harrington 2016). Individuals may also seek out legally obtained tax advantages. In the UK, it was possible until 2019 to bypass capital gains tax on properties by wrapping them in a foreign entity. Instead of selling the property itself, one could simply sell the shares in the company that held the property, which rendered the transaction a form of “indirect disposal” that was not subject to the standard taxes involved when selling property (see Johannesen et al. 2022). Legal transformations since the 2010s have made it more difficult to gain legitimate tax benefits in the UK via single wrapping. Some of our cases, therefore, are likely to include people who wrapped to gain tax benefits that ceased to exist and simply did not change their structure. Hiding ownership through non‐transparent multi‐wrapping, however, can also supply a means to not legally avoid but rather illegally evade taxes owed.

**FIGURE 3:**
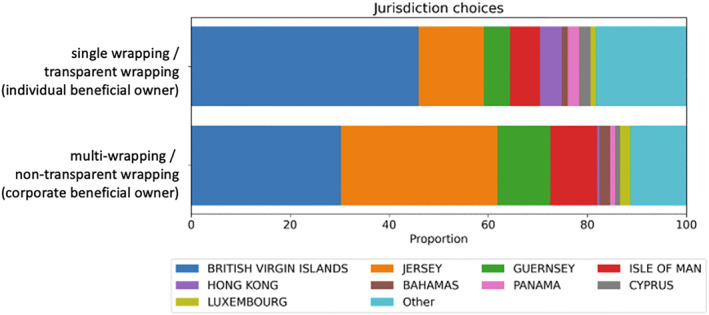
Entry‐layer jurisdictions used in single‐ and multi‐wrapping (2024). Authors' compilations.

When we examine patterns in how offshore layering operates in the high‐end UK property sector, we can specify the distinctiveness of non‐transparent (effectively multi‐wrapping that hides ownership from public reporting requirements) choices by comparing them with transparent wrapping (effectively single wrapping that publicly reveals ownership). As discussed above, the ROE reporting requirements eliminated the de facto secrecy available to individuals, and in a remarkable 52% of cases people declared their offshore ownership (we analyze these in depth elsewhere). People who carry out transparent wrapping—that is, when the wrapping is only one‐layer thick in reporting significance—that select primarily from a small number of jurisdictions for their offshore structuring (Figure 3). Though these are sometimes cast as “secrecy jurisdictions,” their prevalence among individual beneficial owners who have declared themselves to the UK government suggests that more is going on. Significantly BVI appears in pole position as the choice in nearly half of all cases (46%), corroborating its global reputation as *the* jurisdiction for easy‐use shell companies (see also Sharman 2012; Harrington 2016; Palan et al. 2010). At some distance follow the Crown Dependencies of Jersey (13%), Isle of Man (6%), and Guernsey (5%), which together account for a quarter of the cases of single wrapping. The next most popular jurisdiction, Hong Kong, trails at a mere 4%.

However, BVI's dominance shifts when we probe into the jurisdictions that people choose for their entry layer when carrying out non‐transparent wrapping which effectively hides ownership. If there is a corporate beneficial owner—that is, a further offshore entity rather than an individual beneficial owner appears at the action layer—we find that Jersey (32%) is the top choice, followed closely by BVI (30%), with Guernsey (11%) and Isle of Man (9%) trailing. This remarkable difference in BVI's popularity may be due to the greater “substance” possibilities that Jersey offers over BVI, which can facilitate benefits of multi‐layer wrapping as analyzed below. Notably, Jersey supplies additional legal advantages through its popular trust structures, which are regulated and taxed based on the location of the fiduciary (typically a service provider) rather than the location of the beneficial owner. Jersey established its trust structures to leverage lags in UK laws and create benefits vis‐à‐vis UK investments and has continued to adjust to UK law over time (Morriss and Ku 2024). As such, it is an apt choice, from the point of view of beneficial owners, when selecting a jurisdiction to wrap UK properties.

We also find that people who carry out non‐transparent wrapping are more likely to locate their entry‐layer entities in the BVI and Crown Dependencies (82%) than are those who wrap transparently (70%) and in these cases the “other” category is proportionally less. People whose ownership is hidden through non‐transparent wrapping select one of the Crown Dependencies as their entry‐layer jurisdiction in 52% of cases, in contrast to 24% for those who reveal ownership through transparent wrapping. BVI, as we shall see in the following analysis, attracts action‐layer entities from around the world, whereas the Crown Dependencies attract them from themselves and other Crown Dependencies, suggesting the presence of a thicker regional offshore circuit running through them.

**FIGURE 4:**
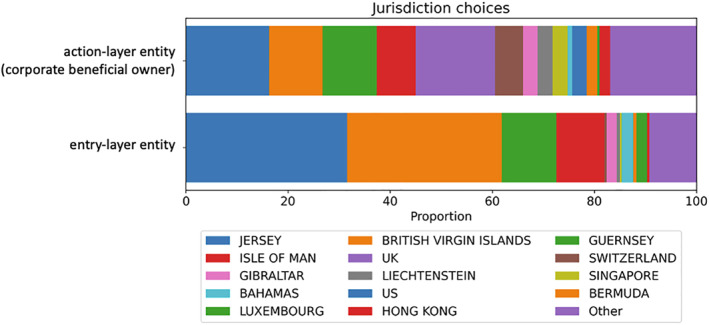
Entry‐ and action‐layer jurisdictions used in non‐transparent/multi‐wrapping (2024). Authors' compilations.

In cases of non‐transparent wrapping, the most common jurisdiction that people select for action‐layer entities remains Jersey (16.3%), followed by the UK (15.5%), Guernsey (11%), BVI (10%), and the Isle of Man (8%). Figure 4 compares the location of the action‐layer entity with that of the entry‐layer entity, taken from Figure 3. The dramatic shift away from BVI is surprising given its reputation as the default incorporation center globally, as the qualitative literature on offshore has shown (see Palan et al. 2010; Sharman 2012; Harrington 2016). The double‐holding within Jersey, we suggest, is due to a selection effect of substance‐driven structuring discussed below.

In over half of all cases, people who wrap non‐transparently choose an action‐layer jursidiction that is geographically local to the property and has legal structures that are integrated with those in the UK. Closely matching Jersey in popularity is the UK itself, a choice that is notable but not startling given the location of the property and the likely location of the service provider making the arrangements, as discussed below. Immediately behind them—and with a similar volume when taken together—are the other Crown Dependencies, which have developed their financial service sectors in response to developments in the UK (Morriss and Ku 2024). Following these leaders at some distance are a smaller set of jurisdictions whose entities hold a handful to a dozen or so expensive properties. The qualitative research on offshore would likely find no surprises on the list, but we are able to capture their magnitude. Notably, the US, the Netherlands, and Australia surface among the choices, although they are often kept off the offshore registers maintained by international or supranational organizations and are rarely incorporated into quantitative work on offshore. Their appearance here suggests the utility of the legal structures that qualitative research has identified as establishing offshore advantages (see Weitzman 2022; Palan et al. 2010).

**FIGURE 5:**
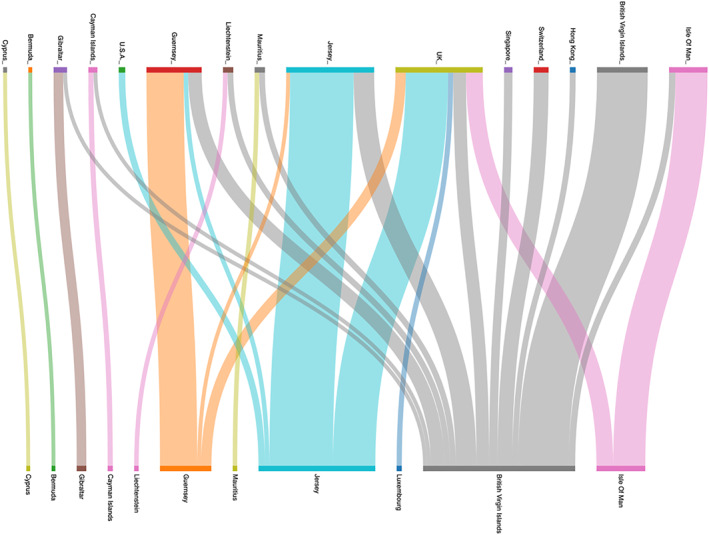
Holding patterns between action‐layer entity jurisdictions (top) and entry‐layer entity jurisdictions (bottom) (2024). Authors' compilations.

Our data also enable us to isolate patterns of connection between the action‐ and entry‐layer entities when the individual owners of the property remain hidden. Figure 5 visualizes these connections and suggests three predominant formations. The first are small flows from a jurisdiction to only itself. The second are small flows from a wide array of action‐layer jurisdictions to a select entry‐layer jurisdiction. The third are large flows from particular places to a select entry‐layer jurisdiction.

**FIGURE 6:**
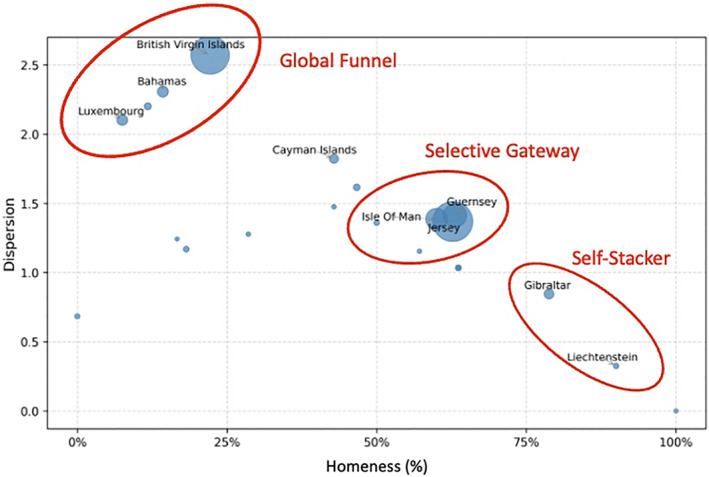
Type of connection between entry‐layer jurisdictions and action‐layer jurisdictions (2024). Authors' compilations. Points are all jurisdictions with at least five distinct entry‐layer entities. Labels indicate jurisdictions with at least 25 distinct entry‐layer entities, except Liechtenstein which has only ten.

These formations are best isolated through an entropy analysis (Figure 6). Entropy, as operationalized here, measures both the diversity and the relative balance of the connections between the action‐ and the entry‐layer entities (see the Methods Section). On the *x*‐axis, “homeness” captures the extent to which a second‐layer entity in a particular jurisdiction reselects its own jurisdiction for the entry‐layer entity. On the *y*‐axis, “dispersion” captures the extent of diversity in the locations of the action‐layer entity that select particular jurisdictions for the entry‐layer entity.

From this we can identify three predominant formations. *Global funnels* tend to have high dispersion (a score of at least 1.5) but low homeness (a score under 50%). If we look at jurisdictions that make our minimum cutoff of five or more entities, we find that 38% of the entry‐layer entities are established in global funnels. BVI is the most substantial example, but the same pattern can be found in the Bahamas and Luxembourg. In such cases, an entry‐layer jurisdiction is likely to be selected by people using action‐layer entities located in a wide range of jurisdictions and with a relatively even distribution of diversity. The result is an octopus‐like structure, as seen with BVI in Figure 5. Effectively, BVI serves as the “go‐to” option for actors around the world to establish an entry‐layer entity to wrap their UK property non‐transparently. Qualitative work suggests that the ease and low cost of incorporation, the protective legal structures, and BVI's reputation for maintaining secrecy are key drivers of this choice (Palan et al. 2010; Sharman 2012; Harrington 2016). It is likely that the Bahamas mirrors these motives. Luxembourg's multi‐directional popularity may be a legacy of UK tax reform that enabled the country to serve as a dodge around regulations for a time. Legislation implemented in 2019 banned the avoidance of UK capital gains tax by wrapping assets in foreign corporation. However, a loophole enabled Luxembourg to continue in this capacity until it was closed by a treaty implemented in 2023 (see Johannesen et al. 2022). Though we cannot see this in our data, which go back to only 2023, it is possible that the loophole widened the attractiveness of using Luxembourg as an entry‐layer wrapper and that the actors did not restructure after these legal changes.

*Selective gateways*, by contrast, enjoy similar popularity among action‐layer entities located both at home and abroad. We define these as jurisdictions with a dispersion score between 1 and 1.5 and a homeness score of between 50 and 75%. This popular form accounts for 54% of entry‐layer entities. The most sizable case in our data is Jersey, though Guernsey and the Isle of Man operate in parallel fashion. These three Crown Dependencies are known for supplying specialist financial services that have developed in response to transformations in UK legal structures (Palan 2013). The clustering is possibly due to the role of modeling and innovation in the region, along with the nimble reactions of these jurisdictions to transformations in the UK that they leverage to increase the size of their financial service sector. These maneuvers have enabled the Crown Dependencies to “climb the value chain” of offshore facilities and offer more substance (Morriss and Ku 2024). This could make them desirable places to locate an entry‐layer wrapper while they also offer sufficiently substantial financial services to make them a desirable location for carrying out more complex multi‐wrapping.

Finally, there are a small number of *self‐stackers* where we find extreme homeness (a score over 75%). Gibraltar and Liechtenstein, alongside Cyprus and Bermuda, are indicative cases of this uncommon pattern, used in only 3% of the entry‐layer entities. In self‐stackers, an action‐layer entity simply creates its entry‐layer entity in the same jurisdiction. However, dispersion is low: these places attract little interest for entry‐layer entity formation from jurisdictions outside them. Although places like Gibraltar and Liechtenstein supply specialist services, they are small players in the space. In examining trends in the legal ecology of offshore, Morriss and Ku (2024) suggest that jurisdictions that do not offer integrated financial services and nor innovate in response to legal transformations elsewhere remain isolates that do not scale. Financial sectors in such places may also prefer to offer expensive and tailored services to a smaller list of very wealthy clients.

**FIGURE 7:**
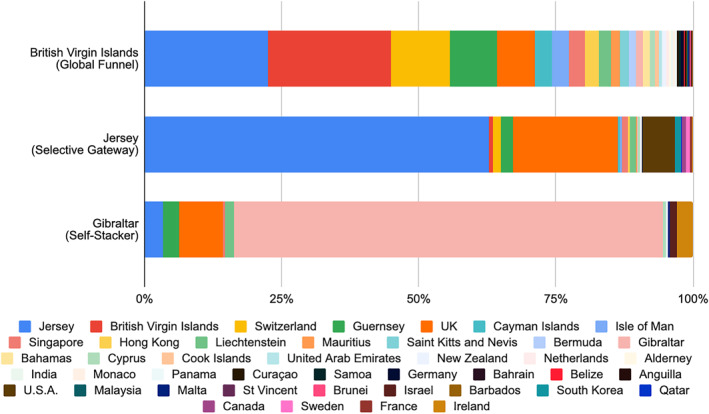
Model cases of each dispersion type (2024). Authors' compilations.

These three multi‐wrapping patterns that hide ownership are made clearest by comparing the top entry‐layer jurisdictions instantiating each type (Figure 7). BVI exhibits a relatively even balance among the action‐layer jurisdictions feeding into it: there may be some leaders, but no place predominates. Jersey, by contrast, sees nearly two thirds of its cases come from within Jersey, followed by the UK at around 19%. Yet it also attracts substantial interest from elsewhere, which accounts for a further 15% of its business. Gibraltar, however, receives the vast majority (77%) of its business from within Gibraltar, with no other jurisdiction reaching even 10%—the next highest is the UK at 8%.

Are there functional differences in these formations? The qualitative literature on offshore gives different possibilities. Some evidence suggests that service providers may select offshore locations based on what fits a client's interests and gets the job done efficiently, and that a wider range of jurisdictions is better when disaggregating assets through complex structures (Harrington 2016). Other research indicates that service providers prefer to work with jurisdictions whose laws they understand well and avoid the costs of learning the ins‐and‐outs of new legal systems (Morriss and Ku 2024). The former points to the utility of brass plate offerings that simply provide convenient and efficient entity formation services; the latter is indicative of decision‐making based on substance, as discussed earlier.

**FIGURE 8:**
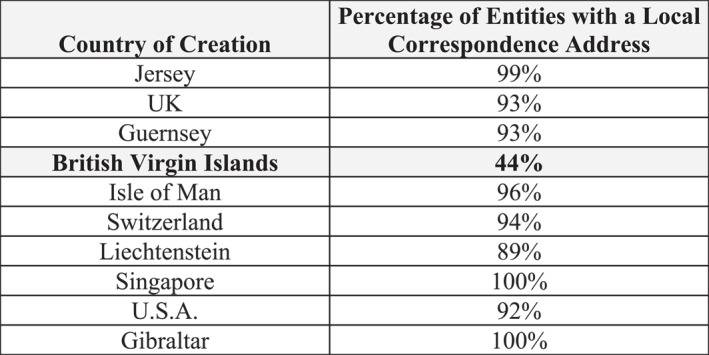
Address alignment of action‐layer entity (2024). Authors' compilations. The figure displays the top ten jurisdictions based on the number of entities incorporated.

Unfortunately, we do not have a direct measure for assessing whether people turn to particular jurisdictions for brass plate or substance services. However, indirect evidence may be gained by examining the correspondence address of the action‐layer entity, or what we term *address alignment* (Figure 8).^7^ If the correspondence address is in a jurisdiction different to the entity's location, then it is likely that the place where the entity was created serves as merely a brass plate and that the location of an entity's management, in reality or projected appearance, is elsewhere. Though we do not have a direct measure of substance, the alignment of the correspondence address and the location of the entity is a reasonable minimum condition for it.

As Figure 8 makes clear, the majority of the action‐layer entities have correspondence addresses in the same jurisdictions where they are located. This is the configuration one would expect if the intermediaries carrying out the work of offshore structuring are based in the place where they are creating their action‐layer entities. The striking exception is BVI. Only 44% of action‐layer entities incorporated there also list the BVI as the place to send correspondences. The results suggest that the BVI serves as a brass plate not only in the entry layer, but also in the action layer. A comparison with the most popular jurisdiction for action‐layer entity formation, Jersey, points in the same direction. Each action‐layer entity in Jersey wraps on average 3.37 entry‐layer entities, suggesting that more complicated structuring that requires greater substance may be involved. In BVI, however, each action‐layer entity wraps on average only 1.37 first‐layer entities, pointing toward single‐use wrappers that are easy to create. We find not only patterned variation in which jurisdictions are used but also evidence of how they may be used differently.

## 3 Conclusion

Breaking new ground, our analysis supplies the first systematic image of two key layers employed in offshore property structuring. By unpacking the connections between the layers of offshore structures, we supply an interstitial analysis of a specific offshore circuit in practice. We use this to identify the jurisdictions chosen, patterns in their interconnections, and possible differences in how they operate, and we suggest why these different patterns have emerged. Previous quantitative research on offshore real estate holdings either captured only the first layer of structuring or sacrificed generalizability for the penetration afforded by data that carry unspecified selection biases. Our dataset offers a compromise between breadth and depth in a world in which systematic data on offshore structuring remains difficult to obtain.

Peering beyond the first layer, we are able to precisely identify three dominant patterns of structuring between the entry and action layers: global funnel, selective gateway, and self‐stacker. We show that global funnels, with high dispersion and low homeness, see most of their wrapping requests come from elsewhere in the world rather than locally. They are popular within this system, accounting for nearly 40% of cases, and it is likely that the ease and low cost of incorporation, alongside protective legal structures, lie behind their attractiveness. BVI, Bahamas, and Luxembourg operate along these lines. Selective gateways, which are popular wrapping destinations for action‐layer entities both at home and abroad, out‐position them and account for just over 50% of cases. Jersey, Guernsey, and the Isle of Man conform to this pattern. Such places are likely to offer more substantial services than global funnels, which may account for the multi‐layering. In the cases explored here, it may be that regional competition has spurred modeling and innovation out of what once were purely brass plate services (Morriss and Ku 2024). Finally, visible too are a small number of self‐stackers, such as Gibraltar and Liechtenstein, where nearly all the entities formed are created by entities located in the same jurisdiction. These appear to operate in a more isolated manner within the system, which we speculate could result from a preference to offer high‐end tailored services.

We employ an inductive, data‐informed strategy to identify offshore locations based on the choices people make in actual practice, an approach that avoids the problems of bias that can arise when using compilations of tax haven and secrecy lists to identify the “where” of offshore (on these issues see Jansky et al. 2022). It also offers greater specificity and rigor than the proxies, such as FDI imbalances or BIS locational data differentials, that quantitative research often uses to identify offshore systems (see, e.g., Haberly and Wojcik 2014; Langenmayr and Zyska 2023; Zucman 2015). Rather than identifying deviations that suggest offshore usage, we can specify the popularity, scale, and inter‐relationship among key jurisdictions in practice. Our approach also facilitates analysis of an ever‐changing field. The full set of data that we use have been available only since 2023 and at present supply only a snapshot. With time, however, further work employing the approach advanced here would be able to adjust to and incorporate regular transformations in preferred jurisdictions, resulting in a more robust conceptualization of offshore structuring around real estate that is better able to capture temporal changes in jurisdictional selection.

Offshore is not a homogenous space and our research provides relatively precise measures the variegations within how particular jurisdictions are used when structuring UK high‐end residential property. Significantly, we show that the top jurisdictional choice for the creating entry‐layer entities differs between owners who identify themselves and those who continue to wrap further in a non‐transparent manner. BVI is favored by individual beneficial owners who publicly register their holdings whereas Jersey is the top choice for beneficial owners who prefer to wrap their assets yet further through an action‐layer entity and thereby hide their ownership. We use indirect evidence from address alignment and wrapping proportions to suggest that people use BVI as a brass‐plate jurisdiction, whereas Jersey, by contrast, is more likely to supply substance (see also Morriss and Ku 2024). It is possible to speculate that individuals who seek basic wrapping advantages simply employ the fast‐and‐easy products offered by BVI, the most popular shell company incorporation center globally (Sharman 2012). By contrast, those who desire more complex, multi‐layered structures that hold more entities are more likely to turn to services providers in jurisdictions that are themselves home to service providers operating in offshore (see also Sharman 2009). Also pointing in this direction is the minor tendency toward what we identify as *self‐stacking*, or for entities in a given action‐layer jurisdictions to re‐select the same jurisdiction for creating the entry‐layer entity.

In presenting the first detailed analysis of two key layers of offshore structures used to hold expensive residential property in the UK, we do not claim to supply a miniature version of an “offshore world” that can be simply scaled up to understand the global system. To do so would be to fundamentally misapprehend how offshore structuring works, which is always a function of standpoint. Offshore operates at the interstices of legal systems and therefore the power of any configuration depends on the matrix of legal structures to which an individual may be beholden. Our analysis takes this seriously by identifying the patterned relationships that emerge *between* layers of offshore structures. As such, it avoids both the methodological limits and the methodological nationalism of research that posits offshore as a *place*—or set of places—rather than field of possibilities that emerges at the interstices of legal systems. Our findings offer a relatively sharp image of how one part of this field operates from the standpoint of expensive residential property in the UK. This can be further employed in comparison with other cases or to sensitize analysts and policymakers to popular forms and strategies of asset structuring.

What do these findings suggest for policymaking? In the first instance, they indicate that beneficial ownership registries, as imperfect as they are, can be remarkably successful in revealing who owns what. Despite the relative ease of structuring to obscure ownership, we find an individual name behind the property in 52% of all cases—names made visible since the 2023 ROE registration requirements. The glass is half‐full. But it is also half‐empty since nearly 30% of the owners remain hidden by a further layer, as we examine here, and possibly many further layers. We show, however, that the locations of these action‐layer entities are limited—and limited, in the main, to jurisdictions still under the political umbrella of the UK to varying degrees. They may be “offshore” to the UK, but their partial sovereignty still furnishes London with some levers of influence. The response rates to the ROE suggest that transparency initiatives that require property owners to report themselves might capture a substantial haul of low‐hanging fruit. If the UK makes a stronger push for the full implementation of transparency initiatives in its dependencies and overseas territories, it may be able to reveal a tranche of beneficial owners behind the action layer. These are likely to be individuals who are concerned more with asset protection than asset cleaning, but represent a significant set nonetheless. For those who aggressively pursue “serious structuring” to hide ownership, the game of whack‐a‐mole will likely continue. For this reason, a different set of tools will be needed to address the larger challenges around illicit finance and money laundering.

## Funding

The research is funded by ESRC grant “Taxing Ghosts” ES/X001342/1.

## Ethics Statement

The project is part of Taxing Ghosts project with LSE ethics approval.

## Conflicts of Interest

The authors declare no conflicts of interest.

## Data Availability

The data that support the findings of this study are available in Companies House at https://www.gov.uk/government/organisations/companies‐house. These data were derived from the following resources available in the public domain: ‐ OCOD, https://www.gov.uk/guidance/hm‐land‐registry‐overseas‐companies‐that‐own‐property‐in‐england‐and‐wales.

## Endnotes

^1^

https://atlas‐offshore‐world.org/.

^2^
We do not carry out a full legal analysis of the relationships between the jurisdictions and what they offer individuals. The benefits an actor gains are conditioned by a wide range of additional factors, such as their nationality or nationalities, place or places or residence, among many others. It also requires a fine‐grained analysis of the bilateral and multilateral relationships between legal regimes across the world, which is a substantial undertaking that we leave to legal scholars. The specialists in this field are highly paid international lawyers who themselves often specialize in a handful of countries at most due to the complexity of the work.

^3^
In doing so, it is important to note that we are not arguing that we identify a scaled‐down representation of a wider “offshore world.” The ecology of offshore is not a single landscape that exists independently of a particular standpoint but is fundamentally relational. Offshore operates based on the advantages generated in the interstices of specific legal systems meaning that the benefits gained will hinge on the web of juridical obligations that impact a person—whether a human being or a legal being—based on their locational and membership particulars. Whether and how Malta can serve as an offshore financial center for an individual, for example, will depend on whether the person is a Maltese citizen, a citizen of another EU country, or a third‐country national, as well as where they are primarily located or spend time, on top of the type of service at stake. Offshore is positional by definition.

^4^
Systematic analysis of offshore structures and property markets outside the UK are limited, but as far as we have found, they too do not investigate interstitial patterns of offshore structuring. See, for example, Alstadsæter et al. (2022),  (2024) on Agarwal et al. (2021) on Singapore.

^5^
The index is available at a variety of geographic levels, ranging from the national to the local. For each property, we used the most granular index available given the date of the purchase and the address of the property. This was usually the local level, but in some cases for properties transacted in the twentieth century, this was the district or even regional level.
